# The global convergence properties of an adaptive QP-free method without a penalty function or a filter for minimax optimization

**DOI:** 10.1371/journal.pone.0274497

**Published:** 2023-07-10

**Authors:** Ke Su, Shaohua Liu, Wei Lu

**Affiliations:** Key Laboratory of Machine Learning and Computational Intelligence Baoding, College of Mathematics and Information Science, Hebei University, Baoding, China; Northeastern University, China, CHINA

## Abstract

In this paper, we proposed an adaptive QP-free method without a penalty function or a filter for minimax optimization. In each iteration, solved two linear systems of equations constructed from Lagrange multipliers and KKT-conditioned NCP functions. Based on the work set, the computational scale is further reduced. Instead of the filter structure, we adopt a nonmonotonic equilibrium mechanism with an adaptive parameter adjusted according to the result of each iteration. Feasibility of the algorithm are given, and the convergence under some assumptions is demonstrated. Numerical results and practical application are reported at the end.

## Introduction

The minimax problem with nonlinear inequality constraints has the following form,

$$\begin{array}{c} \begin{array}{cc} \min_{x\in R^{n}}\;F\left(x\right)\\ \text{s.t.}\;\;\;f_{i}\left(x\right) & \leq 0,i\in \;I_{2}=\left\{l+1,\cdots ,m\right\} \end{array} \end{array}$$ (1)

where $F\left(x\right)=\text{max}\left\{f_{i}\left(x\right)\right\},i\in \;I_{1}=\left\{1,2,\cdots ,l\right\}$.

The minimax problem is a specific class of nonsmooth optimization problems. The general optimization methods cannot be applied directly in (1) because the objective function is nondifferentiable. The common approaches for (1) are gradient sampling methods [1, 2], Cutting Plane Method, and bundle method [3].subgradient methods [4].

Smoothing is one of the most popular classes among all methods for solving nonsmooth problems. There are two main approaches proposed by previous scholars to deal with this problem. First, approximating the non-differential function by a smooth exponential function with parameters (which is also called entropy function). Shor [4] proposed two smoothing algorithms with an active set strategy and a new adaptive parameter update rule. Second, introduce an artificial additional variable to transform the problem into an equivalent nonlinear programming with smooth constraints, as follows

$$\begin{array}{c} \begin{array}{cc} \text{min}\;\;t\;\;\;\;\;\;\;\;\;\;\;\\ \;\;\;\;\;\;\;\;\text{s.t.}\;\;\;f_{i}\left(x\right)-t & \leq 0,i\in \;I_{1}=\left\{1,2,\cdots ,l\right\}\\ f_{i}\left(x\right) & \leq 0,i\in \;I_{2}=\left\{l+1,\cdots ,m\right\}, \end{array} \end{array}$$ (2).

where $f_{i}\left(x\right):R^{n}\to R$ are Lipschitz continuously function; t∈R.

For (2), there are many algorithms can be used such as gradient projection [5], interior point method [6], trust-region [7], sequential quadratic programming(SQP) [8], penalty function [9], filter methods [10] or QP-free method [11], etc.

The extraordinary efficiency of SQP methods in solving nonlinear constrained optimization problems (NLP) has allowed its extension to many other problems, such as minimax problems [8–13]. But the sequences may fail to converge as the initial point lies far from the optimal point in the SQP algorithm. So penalty function methods were proposed by Courant [13] in 1943 to enhance the convergence of the algorithm. The objective function is defined as the sum of the objective function and penalty term in the penalty function method. In [9], Ma gives an exact smooth penalty function method to solve minimax problems with mixed constraints. However, the choice of penalty parameters during the iterative process is complicated. Meantime, the effectiveness of the penalty function method is significantly affected by it.

Fletcher [14] proposed the filter algorithm which can effectively avoid the choice of penalty parameters. It is inspired by the idea of multi-objective programming in 2002, where the objective function and the constraint violation function are considered separately. The combination of the filter and SQP methods has been applied to the minimax problem due to its satisfactory numerical results. [15] gave a trust-region SQP filtering method combining nonmonotonic techniques to solve the unconstrained minimization problem. Luo [10] constructed a new feasible sub-problem based on working sets and incorporated filtering techniques. Although the filter method has good numerical performance, the update of the filter set also faces the problem that the set is getting larger and the computational storage is growing. On the other hand, the feasibility restoration phase is difficult to avoid in the filter method, which more or less increases the computational effort.

To overcome the possible inconsistency in solving the sub-problem and the high computational cost, Panier [16] proposed a QP-free algorithm (SSLE algorithm) for optimization problems with inequality constraints based on the KKT conditions and Newton’s method. Each iteration requires solving two systems of linear equations with the same coefficient matrix and a least-squares subproblem. Global and superlinear convergence are established without the assumption of stationary point isolate. In [11], Jian and Ma presented a new QP-free algorithm for minimax problems according to the unique structure of these problems. [17, 18] proposed two QP-free algorithms for solving constrained optimization problems respectively.

Inspired by the above study, a nonmonotonic QP-free algorithm without a penalty function or a filter is given in this paper for the minimax problem. And the global convergence, as well as the superlinear convergence under some mild conditions, is proved. This algorithm combines the NCP function to solve in each iteration two nonlinear systems of equations with the same coefficient matrix, which can be viewed as a Newton-quasi-interaction perturbation of the primal and dyadic variables of the KKT condition. An adjustable operator is introduced, which changes in each iteration according to the results of the previous iteration, thus changing the degree of influence of the objective function in this mechanism. A nonmonotonic mechanism is used to avoid the Maratos effect. The working set is introduced to reduce the computational effort further.

The paper consists of the following parts. Section 1 introduces the previous methods for solving minimax problem. In section 2, the structure of the work is described. Section 3 discusses the implementation of the algorithm. Section 4 discusses the global convergence and superlinear convergence rate of the algorithm. In section 5, numerical results and practical application are given. The article has been summarized in the final.

## Description of algorithm

### Preliminaries

Define the following notations:

$$X={\left(x^{T},t\right)}^{T}={\left(x_{1},x_{2},\cdots ,t\right)}^{T};$$

$$I=I_{1}\cup I_{2}=\left\{1,\cdots ,l,l+1,\cdots ,m\right\};$$

$$W_{I}\left(X\right)=(\nabla h_{i}\left(X\right),i\in I);$$

$$\xi (X)=\max_{i\in I}\{0,h_{i}\left(X\right)\};$$

$$I\left(X\right)=\{h_{i}\left(X\right)=\xi \left(X\right),i\in I\};$$

$$\Phi_{I}^{k}=\Phi_{I}\left(X^{k},\mu^{k}\right);$$

$$\omega^{k}=\omega \left(X^{k}\right).$$

The following function *h*_*i*_(*X*) is defined to represent the constraint of (2),

$$h_{i}\left(X\right)=\{\begin{array}{cc} f_{i}\left(x\right)-t & ,i\in I_{1}=\left\{1,2,\cdots ,l\right\};\\ f_{i}\left(x\right) & ,i\in I_{2}=\left\{l+1,\cdots ,m\right\}. \end{array}$$

where $h_{i}\left(X\right):R^{n+1}\to R$ are Lipschitz continuously functions.

Let $G\left(X\right)={\left(h_{1},\cdots ,h_{l},h_{l+1},\cdots ,h_{m}\right)}^{T}:R^{n+1}\to R^{m}$.

(2) are equivalent to

$$\begin{array}{c} \begin{array}{cc} \text{min}\;\;\omega (X)\\ \;\;\;\;\;\;\text{s.t.}\;\;\;h_{i}\left(X\right) & \leq 0,i\in I, \end{array} \end{array}$$ (3)

where *ω*(*X*) = *t*.

The Lagrangian function is defined as

$$\begin{array}{c} L\left(X,\mu \right)=\omega \left(X\right)+\sum_{i\in I}\mu_{i}h_{i}\left(X\right), \end{array}$$ (4)

where *μ* = (*μ*_1_, ⋯, *μ*_*l*_, *μ*_*l*+1_, ⋯, *μ*_*m*_)^*T*^ is the multiplier vector.

$\left(\bar{X},\bar{\mu }\right)$ is called the KKT point for (3) if the following conditions hold,

$$\begin{array}{c} \left\{ \begin{array}{c} \nabla L\left(\bar{X},\bar{\mu }\right)={\left(0,\cdots ,0,1\right)}^{T}+\sum_{i\in I}^{m}\bar{\mu_{i}}\nabla h_{i}\left(\bar{X}\right)=0;\\ h_{i}\left(\bar{X}\right)\leq 0,i\in I;\\ {\bar{\mu }}_{i}^{T}h_{i}\left(\bar{X}\right)=0,i\in I;\\ \bar{\mu }\geq 0. \end{array}\right. \end{array}$$ (5)

To construct the system of equations, we introduce the nonlinear complementarity problem (NCP) function, and *φ*(*a*, *b*) is called an NCP function if the following relationship holds,

$$\begin{array}{c} \varphi (a,b)=0\Leftrightarrow ab=0,a\geq 0,b\geq 0. \end{array}$$ (6)

The NCP function is Lipschitz continuous and differentiable except for the origin. Strong semi-smoothness holds at (0, 0). The Fischer-Burmeister NCP function is a simple NCP function with good theoretical properties and numerical performance.

The Fischer-Burmeister NCP function used in this paper has the following structure:

$$\begin{array}{c} \varphi_{\text{FB}}\left(a,b\right)=\sqrt{a^{2}+b^{2}}-a-b. \end{array}$$

So we have

$$\begin{array}{c} \nabla \varphi_{\text{FB}}\left(a,b\right)=\{\begin{array}{c} (\frac{a}{\sqrt{a^{2}+b^{2}}}-1,\frac{b}{\sqrt{a^{2}+b^{2}}}-1),\left(a,b\right)\neq \left(0,0\right);\\ \left(\zeta -1,\nu -1\right)|\zeta^{2}+\nu^{2}=1,\left(a,b\right)=\left(0,0\right). \end{array} \end{array}$$ (7)

Then the NCP function Φ_*i*_ is defined by

$$\begin{array}{c} \Phi_{I}={\left(\phi_{1},\cdots ,\phi_{m}\right)}^{T}, \end{array}$$

where

$$\begin{array}{c} \phi_{i}\left(X,\mu \right)=\varphi_{\text{FB}}{\left(a,b\right)|}_{a=-h_{i}\left(X\right),b=\mu_{i},i\in I}. \end{array}$$

According to (5), define

$$\begin{array}{c} \Phi \left(X,\mu \right)={\left(\nabla_{X}L{\left(X,\mu \right)}^{T},\Phi_{I}^{T}\right)}^{T}. \end{array}$$ (8)

Clearly, KKT condition (5) holds equivalent to Φ(*X*, *μ*) = 0.

Similar to (7), if (*h*_*i*_(*X*), *μ*_*i*_) ≠ (0, 0), then

$$\begin{array}{c} \begin{array}{cc} \nabla_{X}\phi_{i} & =(\frac{-h_{i}\left(X\right)}{\sqrt{{\left(h_{i}\left(X\right)\right)}^{2}+\mu_{i}^{2}}}+1)\nabla h_{i}\left(X\right),i\in I;\\ \nabla_{\mu }\phi_{i} & =(\frac{\mu_{i}}{\sqrt{{\left(h_{i}\left(X\right)\right)}^{2}+\mu_{i}^{2}}}-1)e_{i},i\in I, \end{array} \end{array}$$

where *e*_*i*_ = (0, ⋯, 0, 1, 0, ⋯, 0)^*T*^, and

$$\nabla h_{i}\left(X\right)=\{\begin{array}{c} {\left(\frac{\partial f_{i}}{\partial x_{1}},\cdots ,\frac{\partial f_{i}}{\partial x_{n}},-1\right)}^{T},i\in I_{1};\\ {\left(\frac{\partial f_{i}}{\partial x_{1}},\cdots ,\frac{\partial f_{i}}{\partial x_{n}},0\right)}^{T},i\in I_{2}. \end{array}$$

If (*h*_*i*_(*X*), *μ*_*i*_) = (0, 0), introduce the following notations:

$$\zeta_{j}^{k}=\{\begin{array}{cc} \frac{-h_{i}^{k}\left(X\right)}{\sqrt{{\left(h_{j}^{k}\left(X\right)\right)}^{2}+{\left(\mu_{j}^{k}\right)}^{2}}}+1 & ,(h^{k}\left(X\right),\mu^{k})\neq \left(0,0\right);\\ 1+\frac{\sqrt{2}}{2} & ,else. \end{array}$$

$$\nu_{j}^{k}=\{\begin{array}{cc} \frac{\mu_{i}^{k}}{\sqrt{{\left(h_{j}^{k}\left(X\right)\right)}^{2}+{\left(\mu_{j}^{k}\right)}^{2}}}+1 & ,(h^{k}\left(X\right),\mu^{k})\neq \left(0,0\right);\\ -1+\frac{\sqrt{2}}{2} & ,else. \end{array}$$

It holds that

$$\nabla_{X}\phi_{i}\left(X,\mu \right)=\left(\zeta +1\right)\nabla h_{i}\left(X\right)|-1\leq \zeta \leq 1;$$

$$\nabla_{\mu }\phi_{i}\left(X,\mu \right)=(\zeta -1))|-1\leq \zeta \leq 1.$$

Let $G_{W}^{k}={\left(h_{j}\right)}^{T}:R^{n+1}\to R^{m}$, *j* ∈ *W*. $\nabla G_{W}^{k}$ is the Hessian matrix of $G_{W}^{k}=\left\{h_{i}^{k}|i\in W\right\}$ and *W* is active set.

Define the coefficient matrix *V*^*k*^ according to (8)

$$V^{k}≔(\begin{array}{cc} B^{k} & \nabla G_{W}^{k}\\ diag\left(\zeta_{W}^{k}\right){\left(\nabla G_{W}^{k}\right)}^{T} & diag{\left(\nu_{W}^{k}-c^{k}\right)}^{T} \end{array}),$$

where *B*^*k*^ is a approximation of the Hessian matrix of *L*(*X*, *μ*), and

$$\begin{array}{c} c_{j}^{k}=c\;\text{min}\{1,{\left\|\Phi^{k}\right\|}^{\tau }\} \end{array}$$.

where *c* > 0 and *τ* > 1 are given parameters.

In this work, to increase the convergence and flexibility of the algorithm, we substitute the objective function with the following equation:

$$\begin{array}{c} \Theta \left(X\right)=\omega \left(X\right)+\delta \|\Phi_{I}{\|}^{2}. \end{array}$$ (9)

**Fig 1:**
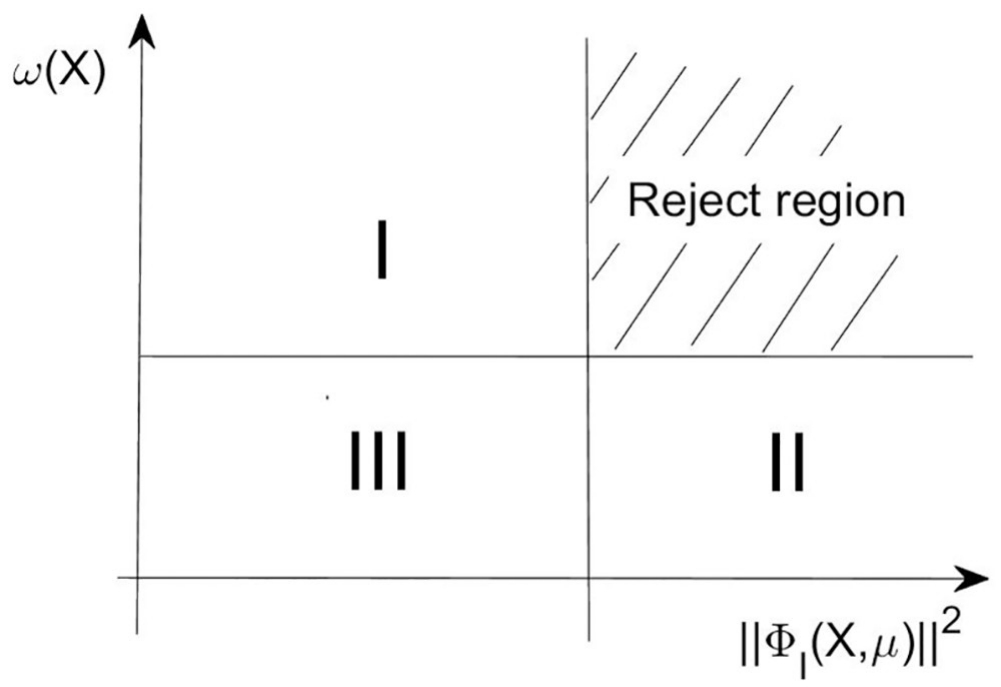
Reject region for iteration points.

We introduce such an adjustable operator, which is not a penalty parameter, but is changed in each iteration based on the effect of the iteration results. Fig 1 shows the initial situation of taking *δ* = 1.

We give the following filter equivalence mechanism:

$$\begin{array}{c} \left\|\Phi_{I}\left({\hat{X}}^{k+1},{\hat{\mu }}^{k+1}\right)\|\leq \theta \max_{0\leq r\leq q(k)-1}\|\Phi_{I}^{k-r}\right\| \end{array}$$ (10)

or both

$$\begin{array}{c} \|\Phi_{I}\left({\hat{X}}^{k+1},{\hat{\mu }}^{k+1}\right)\|\leq \text{max}\{\frac{\iota_{k}+1}{2},\theta_{1}\}h_{max}^{k}; \end{array}$$ (11)

$$\begin{array}{c} \Theta \left({\hat{X}}^{k+1}\right)\leq \Theta \left(X^{k}\right)-\alpha^{k}\theta_{2}\|\Phi_{I}^{k}\|. \end{array}$$ (12)

**Fig 2:**
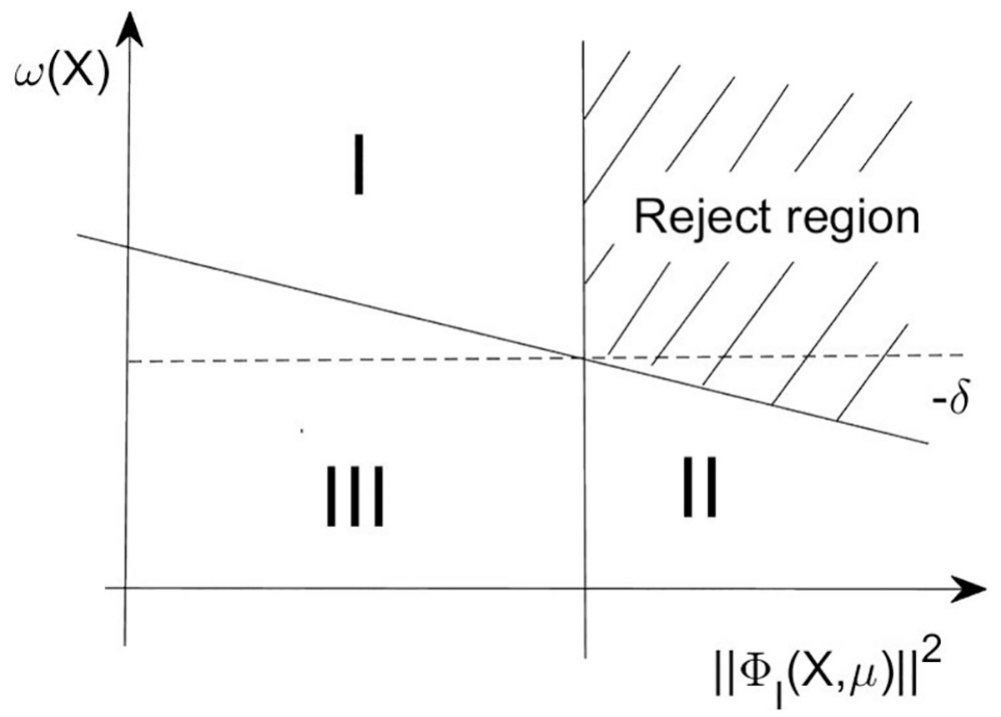
The relaxed reject region for iteration points.

There are three regions in the first quadrant. If the trial point *X*_*k*_ is located in region I at the *k*th iteration, i.e., (10) is satisfied at the current trial point, but (11) and (12) are not satisfied. Then this point is accepted. If the trial point *X*_*k*_ is located in reject region, i.e., the function value and constraint violation are not yielded a satisfactory decrease. So the point is rejected. If the iteration point lies in region II, i.e., (10) is not hold, but (11) and (12) are satisfied. It means that the objective function is improved, but the constraint violation function does not reach the sufficient descent condition, so we need to tighten up our acceptance region (see Fig 2).

So we adjust the parameter *δ*_*k*_ as follows,

$$\begin{array}{c} \delta_{k+1}=\text{min}\{\bar{\delta },\delta_{k}+|\frac{\omega^{k}-\omega^{k+1}}{\|\Phi_{I}^{k}{\|}^{2}-\|\Phi_{I}^{k+1}{\|}^{2}}|\}, \end{array}$$ (13)

where $\bar{\delta }$ is a constant.

**Fig 3:**
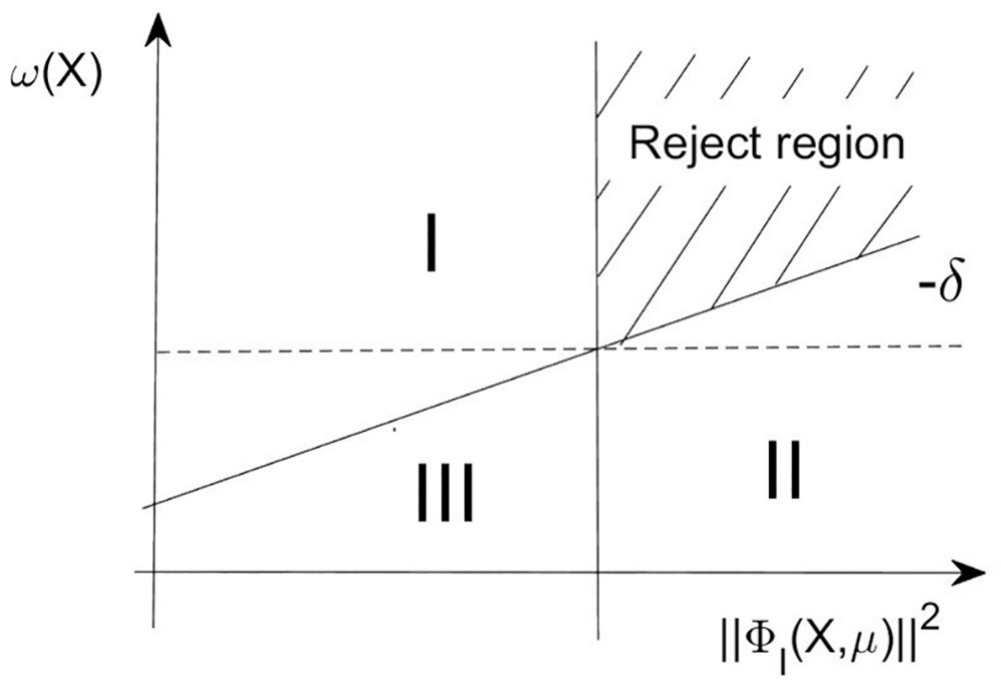
The tightened reject region for iteration points.

If *X*_*k*_ is located in region III, which means the algorithm makes a good improvement in the objective function and the constraint violation function, So we intend to relax the acceptance criteria and expect further improvements. This means increasing the value of *δ*_*k*_ to make the rejected region narrower. (see Fig 3).

Adjust *δ*_*k*_ as follows.

$$\begin{array}{c} \delta_{k+1}=\text{max}\{-\bar{\delta },\delta_{k}-|\frac{\omega^{k}-\omega^{k+1}}{\|\Phi_{I}^{k}{\|}^{2}-\|\Phi_{I}^{k+1}{\|}^{2}}|\}. \end{array}$$ (14)

### Algorithm A

Based on the above analysis, we give algorithm A for the problem (1).

**Step0**: (Initialization.)

Choose an initial point $X^{0}\in R^{n}$;$0<\beta ,\gamma ,\gamma_{1},\gamma_{2},\gamma_{3},\theta ,\theta_{1},\theta_{2},\bar{\delta }<1$;$h_{max}^{0}=0;\iota_{0}=1$,$B^{0}=I\in R^{n\times n}$.

**Step1**: (Working set.)

**Step1.1** Set *i* = 0, *ϵ*_*k*,*i*_ = *ϵ*_*k*−1_.

**Step1.2** Compute $I_{k,i}=\left\{j\in I|-\epsilon_{k,i}\leq h_{j}(X^{k})-\xi (X^{k})\leq 0\right\}$.

**Step1.3** If $det(W_{I_{k,i}}^{T}(X^{k})W_{I_{k,i}}(X^{k}))\geq \epsilon_{k,i}$, set $I_{k}=I_{k,i},W_{k}=W_{I_{k}}(X^{k})$, and *ϵ*_*k*_ = *ϵ*_*k*,*i*_, go to Step 2. Otherwise, let *ϵ*_*k*,*i*+1_ ≔ *βϵ*_*k*,*i*_, let *i* ≔ *i* + 1,go to Step 1.2.

**Step2**:(Search directions.)

**Step2.1** If $\Phi_{I}^{k}\neq 0$, compute $\left(d^{k0},{\bar{\lambda }}^{k0}\right)$ by solving (15) in (*d*, λ):

$$\begin{array}{c} V^{k}(\begin{array}{c} d\\ \lambda \end{array})=(\begin{array}{c} -\nabla \omega^{k}\\ 0 \end{array}). \end{array}$$ (15)

Let

$$\begin{array}{c} \lambda_{j}^{k0}=\{\begin{array}{c} 0,j\in I\backslash I_{k};\\ \lambda_{j}^{k0}=\nu_{j}^{k}{\bar{\lambda }}_{j}^{k0}/(-\nu_{j}^{k}+c_{j}^{k});j\in I,\nu_{j}^{k}\neq 0;\\ \lambda_{j}^{k0}={\bar{\lambda }}_{j}^{k0};j\in I,\nu_{j}^{k}=0. \end{array} \end{array}$$ (16)

**Step2.2** Compute $\left(d^{k1},{\bar{\lambda }}^{k1}\right)$ by solving (17) in (*d*, λ):

$$\begin{array}{c} V^{k}(\begin{array}{c} d\\ \lambda \end{array})=(\begin{array}{c} -\nabla L^{k}\\ -\Phi_{I}^{k} \end{array}). \end{array}$$ (17)

Let

$$\begin{array}{c} \lambda_{j}^{k1}=\{\begin{array}{c} 0,j\in I\backslash I_{k};\\ \lambda_{j}^{k1}=\nu_{j}^{k}{\bar{\lambda }}_{j}^{k1}/(-\nu_{j}^{k}+c_{j}^{k});j\in I,\nu_{j}^{k}\neq 0;\\ \lambda_{j}^{k1}={\bar{\lambda }}_{j}^{k1};j\in I,\nu_{j}^{k}=0. \end{array} \end{array}$$ (18)

If $\Phi_{I}^{k}=0$, then *d*^*k*^ = *d*^*k*0^, λ^*k*^ = λ^*k*0^; else, if *d*^*k*^ = 0, then we let *d*^*k*^ = *d*^*k*1^, λ^*k*^ = λ^*k*1^. If neither is satisfied, then compute *ρ*^*k*^ according to the following definition:

$$\rho_{1}^{k}=\{\begin{array}{c} 1,\left\langle \nabla \omega \left(X^{k}\right),d^{k1}\right\rangle \leq \gamma <\left\langle \nabla \omega^{k},d^{k0}\right\rangle ;\\ \left(1-\gamma \right)\frac{\left\langle \nabla \omega^{k},d^{k0}\right\rangle }{\left\langle \nabla \omega^{k},d^{k0}-d^{k1}\right\rangle },otherwise. \end{array}$$

If $-\gamma_{1}\langle d^{k0},\nabla f^{k}\rangle \geq h_{\text{max}}^{k}$, $\|\Phi_{I}^{k}\|\leq \gamma_{2}h_{\text{max}}^{k}$, then $\rho^{k}=\rho_{1}^{k}$, else *ρ*^*k*^ = *γ*_3_. Denote

$$d^{k}=\left(1-\rho^{k}\right)d^{k0}+\rho^{k}d^{k1};$$

$$\lambda^{k}=\left(1-\rho^{k}\right)\lambda^{k0}+\rho^{k}\lambda^{k1}.$$

**Step3**: (Adjustment nonmonotonous line search.)

**Step3.1** If

$$\begin{array}{c} \|\Phi \left({\hat{X}}^{k+1},{\hat{\mu }}^{k+1}\right)\|\leq \theta \|\Phi \left(X^{k},\mu^{k}\right)\|. \end{array}$$ (19)

and (10) or (12) at least one is satisfied, update *δ*_*k*_ by (14), Let

$$\begin{array}{c} {\hat{X}}^{k+1}=X^{k}+d^{k},{\hat{\mu }}^{k+1}=\mu^{k}+\lambda^{k}. \end{array}$$ (20)

**Step3.2** Let *M* be a non-negative integer, and for each *k* ≥ 1, let *q*(*k*) satisfy

$$\begin{array}{c} q(0)=0,0\leq q(k)\leq \text{min}\{q(k-1)+1,M\}, \end{array}$$

Defined

$$\begin{array}{c} {\hat{X}}^{k+1}=X^{k}+\alpha^{k}d^{k},{\hat{\mu }}^{k+1}=\mu^{k}+\alpha^{k}\lambda^{k}. \end{array}$$ (21)

where *α*^*k*^ = *τ*^*j*^, *j* is the smallest non-negative integer that satisfying

$$\begin{array}{c} \left\|\Phi_{I}\left({\hat{X}}^{k+1},{\hat{\mu }}^{k+1}\right)\|\leq \theta \max_{0\leq r\leq q(k)-1}\|\Phi_{I}^{k-r}\right\| \end{array}$$ (22)

or both

$$\begin{array}{c} \|\Phi_{I}\left({\hat{X}}^{k+1},{\hat{\mu }}^{k+1}\right)\|\leq \text{max}\{\frac{\iota_{k}+1}{2},\theta_{1}\}h_{max}^{k}; \end{array}$$ (23)

$$\begin{array}{c} \Theta \left({\hat{X}}^{k+1}\right)\leq \Theta \left(X^{k}\right)-\alpha^{k}\theta_{2}\|\Phi_{I}^{k}\|. \end{array}$$ (24)

**Step4**: (Update.)

If (10)-(12) hold, update *δ*_*k*_ by (14), and let

$$\begin{array}{c} X^{k+1}={\hat{X}}^{k+1};\mu^{k+1}={\hat{\mu }}^{k+1}. \end{array}$$ (25)

Else, If (10) holds, but (11) and (12) are not all satisfied, then update *X*^*k*^ and *μ*^*k*^ by (25).

Otherwise, update *δ*_*k*_ by (13), and let

$$\begin{array}{c} X^{k+1}=X^{k},\mu^{k+1}=\mu^{k}. \end{array}$$ (26)

Compute *B*^*k*+1^ by BFGS updated formula.

If (10) holds at *X*^*k*+1^ but not at *X*^*k*^, $h_{max}^{k+1}=\Phi_{I}^{k}$; else, $h_{max}^{k+1}=h_{max}$.

If (10) holds, update *ι*_*k*_ by

$$\iota_{k+1}=\underset{0\leq r\leq q(k+1)-1}{\text{max}}\|\Phi_{I}^{k+1-r}\|/\underset{0\leq r\leq q(k)-1}{\text{max}}\|\Phi_{I}^{k-r}\|.$$

Else, *ι*_*k*+1_ = *ι*_*k*_. *k* = *k* + 1, go back to step 1.

**Remark 2.1** The Eqs (10) and (12) are composed of Lagrange multipliers and the KKT condition. The solution of the system satisfies the first-order optimality condition of the original problem.

**Remark 2.2** For convenience, if (10) holds, then the iterative step is called is called Φ-step; if (11) and (12) hold, it is called Θ-step.

**Remark 2.3** Obviously, *δ*_*k*_ is bounded. And the Lagrangian function is Lipschitz continuous.

## Implementation of algorithm

**Assumption A1** *B*^*k*^ is positive definite and there exists positive numbers *m*_1_ and *m*_2_ such that

$$\begin{array}{c} m_{1}{\left\|d\right\|}^{2}\leq d^{T}B^{k}d\leq m_{2}{\left\|d\right\|}^{2} \end{array}$$ (27)

for all $d\in R^{n}$ and all *k*.

**Assumption A2** {*X* ∣ *ω*(*X*) ≤ *ω*(*X*^0^)} and ‖*μ*^*k*^ + λ^*k*^‖ are bounded as *k* is sufficiently large.

**Assumption A3** $\omega \left(X\right):R^{n+1}\to R$ and $h\left(X\right):R^{n+1}\times \Omega \to R$ are Lipschitz continuously differentiable. For all $a,b\in R^{n+m}$,

$$\begin{array}{c} \left\|\nabla \Omega \left(a\right)-\nabla \Omega \left(b\right)\right\|\leq m_{0}\left\|a-b\|,\|\Phi \left(a\right)-\Phi \left(b\right)\right\|\leq m_{0}\left\|a-b\right\|, \end{array}$$

where *m*_0_ > 0 is the Lipschitz constant.

**Assumption A4** The Mangasarian-Fromovitz qualification condition (MFCQ) is satisfied at $X_{i}^{*}$, *i* ∈ *W*(*X*).

**Assumption A5** The sequence of {*B*^*k*^} satisfies

$$\frac{\left\|(B^{k}-\nabla_{X}^{2}L(X^{k},\mu^{k}))d^{k1}\right\|}{\left\|d^{k1}\right\|}\to 0.$$

**Assumption A6** The strict complementarity condition holds at (X*, μ*).

**Remark 3.1** It follows from A3 that the Lagrangian function (4) is Lipschitz continuous.

The following lemmas show the algorithm is well defined.

**Lemma 3.1** Step 1 is finitely terminated.

**proof** Assume that the conclusion is not valid, then step 1 will run an infinite number of times.

$$\begin{array}{c} \det[W_{J_{k,i}\cup L}^{T}W_{J_{k,i}\cup L}]<\frac{1}{2^{i}}\epsilon_{0}. \end{array}$$ (28)

From the definition of *W*_*k*_, we know that *W*_*k*,*i*+1_ ⊆ *W*_*k*,*i*_. As *i* is large enough, *W*_*k*,*i*_ = *W*_*k*,*i*+1_ marked as $W_{k}^{*}$.

Then we have $\det[W_{J_{k,i}\cup L}^{T}W_{J_{k,i}\cup L}]=0$ and $W_{k}^{*}=I\left(X^{k}\right)$.

That is in contradiction to A4.

**Lemma 3.2** If Φ^*k*^ ≠ 0, *V*^*k*^ is nonsingular for all *k*.

**proof** Let *V*^*k*^(*u*^*k*^, *v*^*k*^) = 0, where *u* = (*u*_1_, …, *u*_*m*_)^*T*^,*v* = (*v*_1_, …, *v*_*m*_)^*T*^. From (17), we have

$$\begin{array}{c} B^{k}u+\nabla G_{W}^{k}v=0 \end{array}$$ (29)

$$\begin{array}{c} \text{diag}\left(\zeta_{W}^{k}\right){\left(\nabla G^{k}\right)}^{T}u+\text{diag}{\left(\nu_{W}^{k}\right)}^{T}v=0. \end{array}$$ (30)

Due to Φ^*k*^ ≠ 0, there has $\zeta_{W,j}^{k}\neq 0$ and $\nu_{W,j}^{k}\neq 0$ for any *j* by their definitions.

$$\begin{array}{c} v=-{\left(\text{diag}{\left(\nu_{W}^{k}\right)}^{T}\right)}^{-1}(\text{diag}\left(\zeta_{W}^{k}\right){\left(\nabla G^{k}\right)}^{T}u. \end{array}$$ (31)

Taking (31) to (29), then pre multiplying (29) by *u*^*T*^, we get

$$u^{T}B^{k}u+u^{k}{\left(\nabla G^{k}\right)}^{T})(-{\left(\text{diag}{\left(\nu_{W}^{k}\right)}^{T}\right)}^{-1}\left(\text{diag}\left(\zeta_{W}^{k}\right){\left(\nabla G^{k}\right)}^{T}u\right)=0,$$

where *B*^*k*^ is positive definite, and ${\left(\nabla G^{k}\right)}^{T})(-{\left(\text{diag}{\left(\nu_{W}^{k}\right)}^{T}\right)}^{-1}(\text{diag}\left(\zeta_{W}^{k}\right){\left(\nabla G^{k}\right)}^{T}$is semi-definite.

So we got *u* = 0. Submitting *u* = 0 to (31), then *v* = 0. Since (*u*, *v*) = 0 is the unique solution of *V*^*k*^(*u*^*T*^, *v*^*T*^)^*T*^ = 0, *V*^*k*^ is nonsingular, the conclusion holds.

**Lemma 3.3** If *d*^*k*0^ ≠ 0,then>

$${\left(d^{k0}\right)}^{T}B^{k}d^{k0}\leq -{\left(d^{k0}\right)}^{T}\nabla \omega^{k},$$

where *B*^*k*^ is an approximation of the Hessian matrix of *L*(*X*, *μ*); ∇*ω*^*k*^ is the gradient vector of *ω*(*X*^*k*^, *μ*^*k*^).

**proof** From (15), we have

$$\begin{array}{c} B^{k}d^{k0}+\nabla G_{W}^{k}\lambda^{k0}=-\nabla \omega^{k}; \end{array}$$ (32)

$$\begin{array}{c} \text{diag}(\zeta_{W}^{k}){\left(\nabla G_{W}^{k}\right)}^{T}d^{k0}+\text{diag}(\nu_{W}^{k}-c^{k}){\hat{\lambda }}^{k0}=0. \end{array}$$ (33)

Then

$$\begin{array}{c} {\bar{\lambda }}^{k0}=-{\left(\text{diag}(\nu_{W}^{k}-c^{k})\right)}^{-1}\text{diag}(\zeta_{W}^{k}){\left(\nabla G_{W}^{k}\right)}^{T}d^{k0}. \end{array}$$ (34)

Taking (34) to (32), then

$$\begin{array}{c} \begin{array}{cc} & \;\;{\left(d^{k0}\right)}^{T}\left(B^{k}d^{k0}+\nabla G_{W}^{k}\lambda^{k0}\right)\\ & ={\left(d^{k0}\right)}^{T}B^{k}d^{k0}-{\left(d^{k0}\right)}^{T}\nabla G_{W}^{k}\text{diag}(\zeta_{W}^{k}){\left(\text{diag}(\nu_{W}^{k}-c^{k})\right)}^{-1}{\left(\nabla G_{W}^{k}\right)}^{T}d^{k0}.\\ & =-{\left(d^{k0}\right)}^{T}\nabla \omega^{k}. \end{array} \end{array}$$

The matrix $-\nabla G_{W}^{k}\text{diag}(\zeta_{W}^{k}){\left(\text{diag}\left(\nu_{W}^{k}-c^{k}\right)\right)}^{-1}{\left(\nabla G_{W}^{k}\right)}^{T}d^{k0}$ is positive semi-definite, so we have

$${\left(d^{k0}\right)}^{T}B^{k}d^{k0}\leq -{\left(d^{k0}\right)}^{T}\nabla \omega^{k}.$$

$$\begin{array}{c} \begin{array}{cc} {\left(d^{k}\right)}^{T}\nabla \omega^{k} & =\left(1-\rho^{k}\right){\left(d^{k0}\right)}^{T}\nabla \omega^{k}+\rho^{k}{\left(d^{k1}\right)}^{T}\nabla \omega^{k}\\ & ={\left(d^{k0}\right)}^{T}\nabla \omega^{k}[1-\left(1-\gamma \right)\frac{\left\langle \nabla \omega^{k},d^{k0}\right\rangle }{\left\langle \nabla \omega^{k},d^{k0}-d^{k1}\right\rangle }+\left(1-\gamma \right)\frac{\left\langle \nabla \omega^{k},d^{k0}\right\rangle }{\left\langle \nabla \omega^{k},d^{k0}-d^{k1}\right\rangle }]\\ & =\theta {\left(d^{k0}\right)}^{T}\nabla \omega^{k}\\ & \leq -\theta {\left(d^{k0}\right)}^{T}B^{k}d^{k0}. \end{array} \end{array}$$

Hence the conclusion holds.

**Lemma 3.4** For any 0 < *α* ≤ 1, there exists *t*_1_ > 0 such that

$$\begin{array}{c} {\left\|\Phi_{I}\left(X^{k}+\alpha d^{k0},\mu^{k}+\alpha \lambda^{k0}\right)\right\|}^{2}-{\left\|\Phi_{I}^{k}\right\|}^{2}\leq t_{1}\alpha^{2}{\left\|\left(d^{k0},\lambda^{k0}\right)\right\|}^{2} \end{array}$$ (35)

**proof** If $\Phi_{I}^{k}=0$, then from (31), there exists *t*_1_ > 0 such that for any 0 < *α* ≤ 1,

$$\begin{array}{c} \begin{array}{cc} {\left\|\Phi_{I}\left(X^{k}+\alpha d^{k0},\mu^{k}+\alpha \lambda^{k0}\right)\right\|}^{2} & ={\left\|\Phi_{I}\left(X^{k}+\alpha d^{k0},\mu^{k}+\alpha \lambda^{k0}\right)-\Phi_{I}^{k}\right\|}^{2}\\ & \leq \alpha^{2}m_{2}^{2}{\left\|\left(d^{k0},\lambda^{k0}\right)\right\|}^{2}. \end{array} \end{array}$$

Therefore the conclusion holds for $\Phi_{I}^{k}=0$.

If $\Phi_{I}^{k}=0$, then *φ*_*i*_(0, 0) = 0 indicates

$$\begin{array}{c} \begin{array}{cc} {\left\|\Phi_{I}^{k}+\alpha \left(\text{diag}\left(\zeta^{k}\right){\left(\nabla G_{W}^{k}\right)}^{T}d^{k0}+\text{diag}\left(\nu^{k}\right)\lambda^{k}\right)\right\|}^{2} & ={\left\|\Phi_{I}^{k}\right\|}^{2}\\ +\alpha^{2}\|\text{diag}{\left(\zeta \right)}^{k}){\left(\nabla G_{W}^{k}\right)}^{T}d^{k} & +\text{diag}{\left(\nu \right)}^{k})\lambda^{k}{\|}^{2}, \end{array} \end{array}$$ (36)

where diag(*ζ*^*k*^) and diag(*ν*^*k*^) are the diagonal matrices with diagonal elements $\zeta_{j}^{k0}$ and $\left(\nu_{j}^{k}-cj^{k}\right)$.

So we have

$$\begin{array}{c} {\left\|\Phi_{I}\left(X^{k}+\alpha d^{k0},\mu^{k}+\alpha \lambda^{k0}\right)\right\|}^{2}={\left\|\Phi_{I}^{k}\right\|}^{2}+O\left(\alpha^{2}\right). \end{array}$$ (37)

Hence the conclusion holds.

**Lemma 3.5** If $\Phi_{I}^{k}\neq 0$, for any *ε* > 0, there exists $\bar{\alpha }>0$, such that for any $0<\alpha \leq \bar{\alpha }$,

$$\begin{array}{c} {\left\|\Phi_{I}^{k}\right\|}^{2}-{\left\|\Phi_{I}\left(X^{k}+\alpha d^{k1},\mu^{k}+\alpha \lambda^{k1}\right)\right\|}^{2}\geq \left(2-\varepsilon \right)\alpha {\left\|\Phi_{I}^{k}\right\|}^{2}. \end{array}$$ (38)

**proof** If $\Phi_{I}^{k}\neq 0$, by (SSLE) we have

$$\begin{array}{c} \text{diag}\left(\zeta^{k}\right){\left(\nabla G_{W}^{k}\right)}^{T}d^{k1}+\text{diag}\left(\nu^{k}-c^{k}\right)\lambda^{k1}=-\Phi_{I}^{k}. \end{array}$$ (39)

For any *i* ≠ 0,

$$\begin{array}{c} \varphi_{i}\left(X^{k}+\alpha d^{k1},\mu^{k}+\alpha \lambda^{k1}\right)=\varphi_{i}^{k}+\alpha \left(\text{diag}\left(\zeta^{k}\right){\left(\nabla G_{W}^{k}\right)}^{T},\text{diag}\left(\nu^{k}-c^{k}\right)\lambda^{k1}\right)+O\left(\alpha^{2}\right). \end{array}$$ (40)

By (36) and (40)

$$\begin{array}{c} \begin{array}{cc} {\left\|\Phi_{I}\left(X^{k}+\alpha d^{k0},\mu^{k}+\alpha \lambda^{k0}\right)\right\|}^{2}\\ = & {\left\|\Phi_{I}^{k}\right\|}^{2}+\alpha^{2}{\left\|\text{diag}\left(\zeta^{k}\right){\left(\nabla G_{W}^{k}\right)}^{T}d^{k1}+\text{diag}\left(\nu^{k}-c^{k}\right)\lambda^{k1}\right\|}^{2}\\ + & 2\alpha {\left(\Phi_{I}^{k}\right)}^{T}\left(\text{diag}\left(\zeta^{k}\right){\left(\nabla G^{k}\right)}^{T}d^{k1}+\text{diag}\left(\nu^{k}-c^{k}\right)\lambda^{k1}\right)+O\left(\alpha^{2}\right). \end{array} \end{array}$$ (41)

Since $c_{i}^{k}\neq 0$, by the definitions of $c_{i}^{k}$ and $\nu_{i}^{k}$ and $\phi_{i}^{k}=0$, the following equation holds

$$\begin{array}{c} \begin{array}{c} {\left\|\Phi_{I}^{k}+\alpha \left(\text{diag}\left(\zeta^{k}\right){\left(\nabla G_{W}^{k}\right)}^{T}d^{k1}+\text{diag}\left(\nu^{k}-c^{k}\right)\lambda^{k1}\right)\right\|}^{2}=\left(1-2\alpha \right){\left\|\Phi_{I}^{k}\right\|}^{2}\\ +\alpha^{2}{\left\|\text{diag}\left(\zeta^{k}\right){\left(\nabla G_{W}^{k}\right)}^{T}d^{k1}+\text{diag}\left(\nu^{k}-c^{k}\right)\lambda^{k1}\right\|}^{2}. \end{array} \end{array}$$ (42)

By (41) and (42), given any *ε* > 0, there is $\bar{\alpha }>0$ such that, for any $0<\alpha \leq \bar{\alpha }$,

$$\begin{array}{c} {\left\|\Phi_{I}^{k}\right\|}^{2}-{\left\|\Phi_{I}\left(X^{k}+\alpha d^{k1},\mu^{k}+\alpha \lambda^{k1}\right)\right\|}^{2}\geq \left(2-\varepsilon \right)\alpha {\left\|\Phi_{I}^{k}\right\|}^{2}. \end{array}$$ (43)

Hence the conclusion holds.

From Lemmas 3.4–3.5, we can obtain the following Lemma 3.6.

**Lemma 3.6** If $\Phi_{I}^{k}\neq 0,$ then given any *ε* > 0, there exists $\bar{\alpha }>0$ such that, for any $0<\alpha \leq \bar{\alpha }$,

$$\begin{array}{c} {\left\|\Phi_{I}^{k}\right\|}^{2}-{\left\|\Phi_{I}\left(X^{k}+\alpha d^{k},\mu^{k}+\alpha \lambda^{k}\right)\right\|}^{2}\geq \rho^{k}\alpha {\left\|\Phi_{I}^{k}\right\|}^{2}. \end{array}$$ (44)

**Theorem 3.1** If Algorithm A does not terminate at *X*^*k*^, then there exits an *α*_*min*_ > 0, such to *α*_*k*_ ≥ *α*_*min*_ > 0, we have either (10) holds, or both (11) and (12) hold.

**proof** If *ρ*^*k*^ = *γ*_3_, for all *k*, $\Phi_{I}^{k}\neq 0$ and any $\alpha \leq \text{min}\{\left(1-\theta \right)/\gamma_{3},\bar{\alpha }\}$, there have

$$\begin{array}{c} {\left\|\Phi_{I}\left(X^{k}+\alpha d^{k},\mu^{k}+\alpha \lambda^{k}\right)\right\|}^{2}\leq \left(1-\rho^{k}\alpha \right)\|\Phi^{k}\|\leq \theta {\left\|\Phi_{1}^{k}\right\|}^{2}\leq \theta \max_{0\leq r\leq m\left(k\right)-1}\|\Phi_{I}^{k-r}\|. \end{array}$$

So (10) holds.

If $\rho^{k}=\rho_{1}^{k}$, by the definition of *ρ*^*k*^, $-\gamma_{1}{\left(d^{k0}\right)}^{T}\nabla \omega^{k}\geq h_{\text{max}}^{k}$, $\|\Phi_{I}^{k}\|\leq \theta h_{\text{max}}^{k}$. From Lemma 6, define $\bar{\delta }>\frac{1}{\gamma_{3}}-\theta_{2}$, for all sufficiently large *k*, we have

$$\begin{array}{c} \begin{array}{cc} \Theta \left(X^{k}\right)-\Theta \left(X^{k+1}\right) & \geq -\alpha^{k}{\left(d^{k}\right)}^{T}\nabla \omega^{k}+\delta_{k}\|\Phi_{I}^{k}\|-\delta_{k+1}\left\|\Phi_{I}^{k+1}\right\|\\ & \geq -\alpha^{k}\gamma_{1}{\left(d^{k0}\right)}^{T}\nabla \omega^{k}-\bar{\delta }\left(\right\|\Phi_{I}^{k}\left\|-\right\|\Phi_{I}^{k+1}\left\|\right)\\ & \geq \alpha^{k}h_{max}^{k}-\bar{\delta }\alpha \rho^{k}\left\|\Phi_{I}^{k}\right\|\\ & \geq \alpha^{k}\theta_{2}\left\|\Phi_{I}^{k}\right\|, \end{array} \end{array}$$ (45)

and

$${\left\|\Phi_{I}\left(X^{k}+\alpha d^{k},\mu^{k}+\alpha \lambda^{k}\right)\right\|}^{2}\leq \theta_{1}{\left\|\Phi_{1}^{k}\right\|}^{2}\leq \gamma_{1}h_{\text{max}}^{k}.$$

The proof is complete.

## Convergence of algorithm

In this section, the global and superlinear convergence rates of this algorithm have been discussed.

If Φ^*k*^ = 0, then (*X*^*k*^, *μ*^*k*^) is KKT point. From Lemma 3.3, if *d*^*k*0^ ≠ 0, then *d*^*k*^ is the decreasing direction of *ω*^*k*^, (*d*^*k*^, λ^*k*^) is the decreasing direction of $\left\|\Phi_{I}^{k}\right\|$. If $\Phi_{I}^{k}=0$ and *d*^*k*0^ = 0, (*X*^*k*^, *μ*^*k*^) satisfies the KKT condition. In the following, without loss of generality, it is assumed that the algorithm does not terminate finitely.

**Lemma 4.1** Assume A1-A4 hold. Φ_*I*_(*X*^*k*^, *μ*^*k*^) → 0, as *k* is large enough.

**proof** Case 1. If (10) holds for all *k* sufficiently large, one has

$$\begin{array}{c} \begin{array}{cc} \left\|\Phi_{I}^{l\left(k+1\right)}\right\| & \leq \max_{0\leq r\leq q\left(k\right)}\|\Phi_{I}^{k+1-r}\|\\ & =\text{max}\left\{\Phi_{I}^{k+1},\Phi_{I}^{l\left(k\right)}\right\}\leq \|\Phi_{I}^{l\left(k\right)}\|. \end{array} \end{array}$$

The sequence$\left\|\Phi_{I}^{l\left(k\right)}\right\|$ is monotone decreasing, which implies it is convergent. From (10), we obtain

$$\|\Phi_{I}\left(X^{k+1},\mu^{k+1}\right)\|\leq \theta_{1}\left\|\Phi_{I}^{l\left(k\right)}\right\|.$$

So {‖Φ_*I*_(*X*^*k*+1^, *μ*^*k*+1^)‖} is convergent. Together with *θ*_1_ ∈ (0, 1), we have

$$\|\Phi_{I}\left(X^{k+1},\mu^{k+1}\right)\left\|\leq \right\|\Phi_{I}^{l\left(k\right)}\|\to 0\left(k\to \infty \right),$$

which illustrates Φ_*I*_(*X*^*k*^, *μ*^*k*^) → 0.

case 2. If (11) and (43) hold for all *k* sufficiently large, we prove the conclusion by contradiction. Then there exists *ϵ*_1_ > 0, such that

$$\begin{array}{c} \begin{array}{c} \|\Phi_{I}\left(X^{k+1},\mu^{k+1}\right)\|\geq \varepsilon >0,\\ h_{max}^{k}\geq \varepsilon >0. \end{array} \end{array}$$

and

$$\begin{array}{c} \Theta \left(X^{k}\right)-\Theta \left(X^{k+1}\right)=\omega^{k}+\delta_{k}\|\Phi_{I}^{k}{\|}^{2}-(\omega^{k+1}+\delta_{k+1}\|\Phi_{I}^{k+1}{\|}^{2}). \end{array}$$ (46)

By (12), (43) and the definition of *θ* and $\bar{\delta }$, we have

$$\begin{array}{c} \begin{array}{cc} \omega^{k}-\omega^{k+1} & \geq \alpha^{k}\theta_{2}\|\Phi_{I}^{k}\|-\bar{\delta }\left(\right\|\Phi_{I}^{k}{\|}^{2}-\|\Phi_{I}^{k+1}{\|}^{2})\\ & \geq \alpha^{k}\theta_{2}\epsilon_{1}-\bar{\delta }\epsilon_{1}\alpha^{k}>0. \end{array} \end{array}$$

Because {*ω*^*k*^} is monotonically decreasing. Then *ω*^*k*^ → −∞ as *k* → +∞ which is contradictory to the hypothesis.

Case 3. If the Φ-step and Θ-step iterative appear alternately.

According to Remark 2.2, the iterations from *k*_*t*_ to *k*_*t*+1_ and *k*_*t*+2_ + 1 to *k*_*t*+3_ are Θ-steps. And the iteration from *k*_*t*+1_ + 1 to *k*_*t*+2_ iteration are Φ-steps.

$h_{\text{max}}^{k}$ is updated only when the transition from the Θ-step to the Φ-step, which indicates that the constraint violation is still large enough to decrease. From step 4, we know if $h_{\text{max}}^{k+1}$ is updated, then

$$\begin{array}{c} \begin{array}{c} h_{\text{max}}^{k+1}=\theta_{1}\|\Phi_{I}^{l\left(k+1\right)}\|\leq \theta_{1}\left\|\Phi_{I}^{l\left(k+1\right)}\right\|=\theta_{1}h_{\text{max}}^{l\left(k\right)}\leq h_{\text{max}}^{k}. \end{array} \end{array}$$

Thus $h_{\text{max}}^{k}\left(X\right)$ is monotonous descent and $\|\Phi_{I}^{k}\left\|\leq \right\|\Phi_{I}^{k+1}\|\leq h_{\text{max}}^{k}$. Meantime, we have

$$\begin{array}{c} \begin{array}{c} h_{\text{max}}^{k_{t}}=h_{\text{max}}^{k_{t}+1}=\cdots ,=h_{\text{max}}^{k_{t+1}};\\ h_{\text{max}}^{k_{t+1}+1}=\max_{0\leq r\leq q\left(k_{t+1}+1-r\right)-1}\left\|\Phi_{I}^{k_{t+1}+1}\right\|=h_{\text{max}}^{k_{t+1}+2}=\cdots =h_{\text{max}}^{k_{t+2}}=\cdots =h_{\text{max}}^{k_{t+3}}. \end{array} \end{array}$$

Donate $\text{max}\{\left(r_{k}+1\right)/2,\theta_{1}\}=\bar{\theta }$, then we have

$$\begin{array}{c} \begin{array}{c} h_{\text{max}}^{k_{t+1}+1}\leq \max_{0\leq r\leq q\left(k_{t+2-r}\right)-1}\bar{\theta }h_{\text{max}}^{k_{t+1}+1-r}\leq \theta_{1}h_{\text{max}}^{k_{t+1}}. \end{array} \end{array}$$

The sequence $\left\{\cdots ,h_{\text{max}}^{k_{t}+1},\cdots ,h_{\text{max}}^{k_{t+2}},\cdots ,h_{\text{max}}^{k_{t+3}},\cdots \right\}$ is monotonous descent for all *α* ≥ 0, and

$$h_{\text{max}}^{k_{t+1}+1}/h_{\text{max}}^{k_{t}+1}=\bar{\theta }<1.$$

So $h_{\text{max}}^{k_{t+1}+1}\to 0,t\to \infty$. Considering the non-negativity of $h_{\text{max}}^{k}$, we have $h_{\text{max}}^{k}\to 0,k\to \infty$. Along with (19), $\|\Phi_{I}^{k}\|\leq \text{max}\{\theta_{1},\bar{\theta }\}h_{\text{max}}^{k}$. Therefore $\lim_{k\to \infty }\|\Phi_{1}^{k}\|=0$.

**Lemma 4.2** Assume A1-A4 hold. If *k* is large enough, then *d*^*k*0^ → **0**, *d*^*k*^ → **0**.

The proof is similar to Lemma 5.4 in [20].

With Lemma 4.1 and 4.2, we can obtain the global convergence of the algorithm.

**Theorem 4.1** The accumlation point (X*, μ*) of the sequence {(*X*^*k*^, *μ*^*k*^)} is the KKT point pair of problem (15).

The convergence rate of the algorithm is discussed next. To make the algorithm converge superlinear, assumptions 5–6 are added.

**Remark 4.1** A5 illustrates (*X*^*k*^, *μ*^*k*^) is a Newton direction with a high order perturbation. A6 shows that Φ is continuously differentiable at each KKT point (X*, μ*).

**Lemma 4.3** The above assumptions hold, then {‖(*V*^*k*^)^−1^‖} and $\left\{\|{\left({\hat{V}}^{k}\right)}^{-1}\|\right\}$ are uniformly bounded. The accumulation matrix V* of {*V*^*k*^} is nonsingular.

The proof is similar to Lemma 5.5 in [19].

Lemma 4.3 implies that the Theorem 4.2 holds.

**Theorem 4.2** Assume A1-A6 hold, the algorithm is superlinearly convergent, i.e., (*X*^*k*^, *μ*^*k*^) converges to (X*, μ*) superlinearly.

## Numerical results

In this section, the numerical results are shown. Problem 1 the section is taken from [19]. The BFGS formula proposed by Broyden et al. is used to update *B*^*k*+1^ as [20].

Tables 1 and 2 show the numerical experimental results of two test questions, where NIT is the number of iterations, NF is the number of times the objective and constraint functions are computed, and NG is the number of times the gradient is computed. and TIME(Unit in seconds) is the CPU runtime. The numerical experiments were computed by Matlab R2020 on a computer with 16.0GB RAM and Intel 11th Gen Intel(R) Core(TM) i5–11320H @ 3.20GHz.

**Table 1:** Test problem 1.

*n*	*NIT*	*NF*	*NG*	*TIME(s)*
2	2	2	2	0.045
3	9	9	16	0.079
4	8	71	78	0.107
5	9	88	96	0.109
6	9	88	96	0.113
7	8	103	110	0.127
8	9	92	100	0.131

**Table 2:** Test problem 2.

*n*	*NIT*	*NF*	*NG*	*TIME(s)*
3	7	38	44	0.083
4	4	19	22	0.065
5	13	172	184	0.173

The parameters involved in the algorithm are chosen as follows: $B^{0}=I\in R^{n\times n}$,*τ* = 2.5, *c* = 0.2, $\bar{\delta }=2$, *γ* = 0.5, *γ*_1_ = 0.1, *γ*_2_ = 0.95, *γ*_3_ = 0.5, *θ* = 0.9, *θ*_1_ = 0.95, *θ*_2_ = 0.1, $h_{max}^{0}=0$, *ι*_0_ = 1,*μ* = [1, 0, …, 0, 1].

**Test problem 1** [21].

$$\begin{array}{cc} \text{Figure}1\;\text{min} & F\left(x\right)\\ s.t. & f_{i}\left(x\right)=\sum_{i=1}^{n-1}\left(x_{i}^{2}+x_{i+1}^{2}+x_{i}x_{i+1}-1\right), \end{array}$$

where

$$\begin{array}{c} F\left(x\right)=\text{max}\{f_{i}\left(x\right)=x_{i}^{2},i=1,\ldots ,n\}. \end{array}$$

**Test problem 2** [21].

$$\begin{array}{cc} \text{min} & F\left(x\right)\\ s.t. & f_{i}\left(x\right)=\left(3.0-0.5x_{i+1}\right)x_{i+1}-x_{i}-2.0x_{i+2}+1,\;i\in \left[1,n-2\right], \end{array}$$

where

$$\begin{array}{c} F\left(x\right)=\text{max}\{f_{i}\left(x\right)=x_{i}^{2},i=1,\ldots ,n\}. \end{array}$$

Then we discuss the application of algorithms to investment portfolios. In the investment problem proposed by Markowitz, there are two objective functions to be considered. One is to maximize the return of the portfolio, and the other is to reduce the risk. In the traditional model, the latter is to minimize the risk (variance) of a set of feasible portfolios for a given level of expected returns. By varying the expected return level as the two objectives for a set of nondominant portfolios, the efficient frontier on returns is determined by the variance and average of the yields in the Markowitz model. Investors can get a suitable portfolio by analyzing the expected investment and return.

In the traditional Markowitz mean-variance model, it is assumed that the investor has some wealth and is ready to invest in a set of securities, which is recorded as a set *P*. *R*_*k*_ represents the return value of each security *k*, which is a random variable. The mean value of *R*_*k*_ can be calculated from historical data. Define the expected return of a security as *μ*_*k*_ = *E*(*R*_*k*_), *k* = 1, ⋯, *P*. *x*_*k*_ is the proportion allocated to a certain security. The weight vector *x*_*k*_ needs to satisfy the following constraints,

$$\sum_{k=1}^{P}x_{k}e^{T}x=1$$

where *e* = {1, ⋯, 1}^*T*^ ∈ *R*^*P*^.

The expected returns of the portfolio are as follows:

$$\begin{array}{c} E\left(R\right)=E\left(x_{k}R_{1}+\cdots +x_{P}R_{P}\right)=x_{1}\mu_{1}+\cdots x_{P}\mu_{P}=\mu^{T}x \end{array}$$

where *μ* = (*μ*_1_, ⋯, *μ*_*P*_)^*T*^.

The variance of the portfolio is

$$\begin{array}{c} \nu \left(R\right)=E\left({\left[\sum_{k=1}^{P}x_{k}R_{k}-E\left(\sum_{k=1}^{P}x_{k}R_{k}\right)\right]}^{2}\right)\\ =\sum_{k=1}^{P}\sum_{j=1}^{P}E[\left(R_{k}-\mu_{k}\right)\left(R_{j}-\mu_{j}\right)]x_{k}x_{j} \end{array}$$

where covariance matrix *Q* = {*Q*_*i*,*j*_} is a symmetric positive semi-definite matrix with uncertain information. Assume that the short-term investment value is uncertain, but there are obviously *x*_*k*_ ≥ 0, *k* = 1, ⋯, *n*. we let *B*_*k*_ and *A*_*k*_ be the upper and lower bounds for *x*_*k*_, that is, *B*_*k*_ ≤ *x*_*k*_ ≤ *A*_*k*_, *k* = 1, ⋯, *P*.

According to the above definition, the base/mean-variance dual-objective optimization problem [22] can be described as

$$\begin{array}{cc} \underset{x\in R^{P}}{\text{min}} & -\frac{\mu^{T}x}{x^{T}Qx}\\ \underset{x\in R^{P}}{\text{min}} & T\left(x\right)\\ \;\text{s.t.}\; & e^{T}x=1,\\ & B_{k}\leq x_{k}\leq A_{k},k=1,\cdots ,P, \end{array}$$

where $T\left(x\right)={\left\|x\right\|}_{0}=\sum_{k=1}^{P}\text{sign}\left(|x_{k}|\right)$, and

$$\begin{array}{c} \text{sign}=\{\begin{array}{cc} -1, & x_{k}<0,\\ 0, & x_{k}=0,\\ 1, & x_{k}>0. \end{array} \end{array}$$

Note that *sign*(‖*x*_*k*_‖) is discontinuous, so we introduce the following approximation function which is locally lipschitz continuity,

$$\begin{array}{c} w_{k}\left(x_{k},\delta \right)=\{\begin{array}{cc} \frac{\delta }{x_{k}-1+\delta }+\delta +1, & x_{k}\leq -\delta ,\\ \frac{\left|x_{k}\right|}{\delta }, & |x_{k}|\leq \delta ,\\ \frac{-\delta }{x_{k}-1+\delta }+\delta +1, & x_{k}\geq \delta , \end{array} \end{array}$$ (47)

where *δ* > 0. For any *x*_*k*_ ∈ *R*, $\lim_{\delta \to 0^{+}}\omega \left(x_{k},\delta \right)=sign\left(\right\|x_{k}\left\|\right)$.

Let *y* = (*x*, *y*_*P*+1_)^*T*^ ∈ *R*^*P*+1^, we get the continuous approximation problem as follows:

$$\begin{array}{c} \begin{array}{cc} & \underset{y\in R^{P+1}}{\text{min}}w\left(y\right)={\left(w_{1}\left(x_{1},\delta \right),\cdots ,w_{P}\left(x_{P},\delta \right),y_{P+1}\right)}^{T}\\ & \;\text{s.t.}\;-\frac{\mu^{T}x}{x^{T}Qx},\\ & \;\;e^{T}x=1,\\ & \;\;B_{k}\leq x_{k}\leq A_{k},k=1,\cdots ,P. \end{array} \end{array}$$ (48)

where **w**(*y*) = (*ω*_1_(*x*_1_, *δ*), ⋯, *ω*(*x*_*p*_, *δ*), *y*_*P*+1_)^*T*^. (48) can be regarded as a minimax problem, which can be solved using the algorithm 1. We take the case of *P* = 2, the optimal portfolio obtained is [0.5;0.5]. This result illustrates that under the condition that the risk and return of each stock are equal, the proportion of each stock in the optimal strategy is determined by the investor’s investment willingness.

## Conclusion

In this paper, we give the properties of global convergence and the global convergence of an adaptive QP-free method for solving the minimization problem. Combining the NCP function and the multiplier, in each iteration two systems of linear equations with the same coefficient matrix are solved, which can be viewed as a perturbation of the primal Variables and dual variables of the KKT condition by the Newton-quasi interaction. A new filter substitution mechanism is given, which retains the advantages of the filter method, avoids the selection of penalty parameters, and eliminates potential storage problems that may arise from the filter. And the objective function is tuned by introducing a flexible operator. A non-monotonic mechanism is used to avoid the Maratos effect to some extent, and the introduction of working set further reduces the workload. The effectiveness and convergence of the intensity algorithm are demonstrated under the assumption of no stability point isolation.
